# Supplementary material for: Ionic transport through a protein nanopore: a Coarse-Grained Molecular Dynamics Study
Source: Sci Rep. 2019 Oct 31;9:15740. doi: 10.1038/s41598-019-51942-y (PMC6823379; doi:10.1038/s41598-019-51942-y)
Supplement: Supplementary file 1 — Supplementary Information [file 41598_2019_51942_MOESM1_ESM.pdf]

Ionic transport through a protein nanopore: a Coarse-Grained Molecular Dynamics Study

Nathalie Basdevant[1], **Delphine Dessaux**[1], **and Rosa Ramirez**[1,*]

[1]LAMBE, Univ Evry, CNRS, CEA, Université Paris-Saclay, 91025, Evry, France
[*]rosa.ramirez@univ-evry.fr

Supplementary Information

In the original MARTINI article, a CG ion is presumed to contain a first hydration shell composed by six water molecules. This number is commonly used to set the ratio between PW molecules and ions for a given molar concentration, although for concentration setting purposes, the 4 to 1 standard MARTINI mapping seems to be a better choice. In our NPT simulations, we have computed this ratio together with the molar concentration in the bulk regions. In our NVT simulations, we will consider that one CG ion represents an all-atom ion with three water molecules (see Fig. S1).

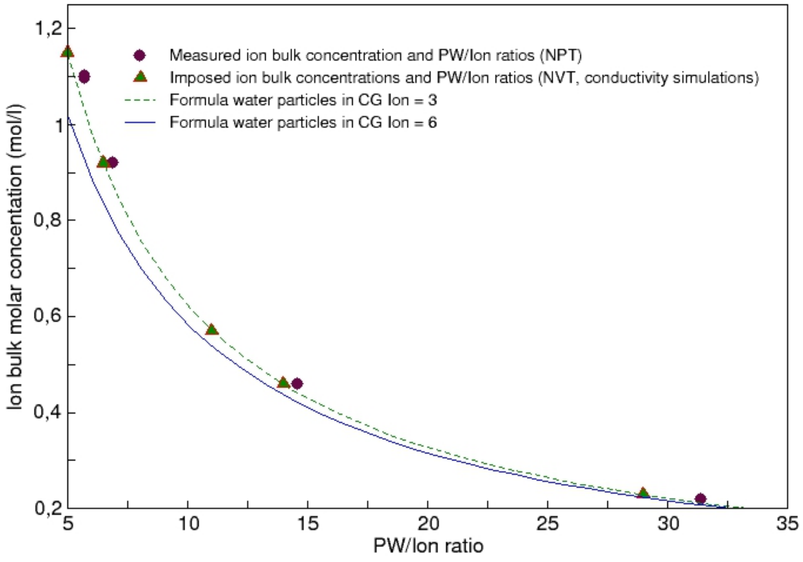

Figure S1. Bulk molar concentration vs. PW/Ion ratio. Circles correspond to measured concentrations in our NPT simulations and triangles correspond to fixed concentrations in our NVT simulations. The dashed line is the theoretical value of molar concentration assuming one CG ion represents an ion plus three AA water molecules. The solid line is the same theoretical value, when a CG ion represents a ion plus six AA water molecules.

The instant current was calculated computing the number of cations and anions crossing a *xy* plane perpendicular to the z axis, with z abscissa equal to the position of the center of mass of the groups in the *trans* end of the pore, colored in blue in the figure S2.

We have defined the "stem" as a region inside the pore going from the z abscissa defined as the "trans" to 4 nm above. It is colored in mauve in the figure.

The evolution of the number of ions inside the stem for different positive and negative biases is represented in figure S3. The asymmetry between positive and negative bias can be observed. We have estimated to 1 μs the time to get to a stationary state.

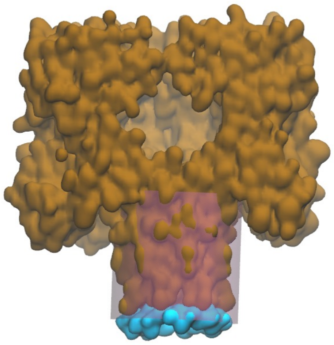

Figure S2. Partial section of α-hemolysin. The group in the *trans* side of the pore (*cyan*), composed of Asp127, Asp128, Thr129, Gly130 and Lys131, is used to define the plan position for the current calculations. The evolution of the number of ions was computed inside the restricted cylinder of 4 nm in height and width (*mauve*) surrounding the stem.

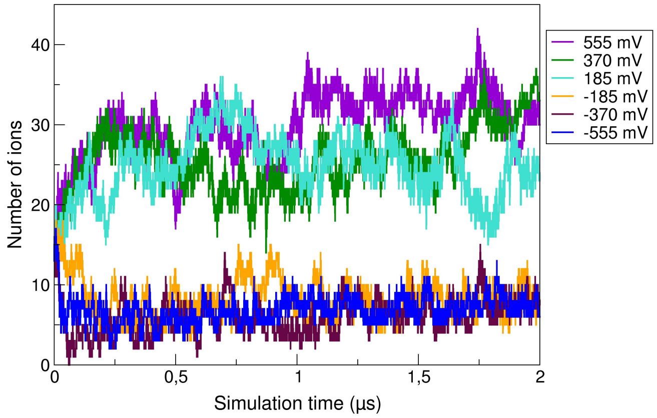

Figure S3. Number of ions in the stem region of the pore along time for different external electric fields applied on the system.

During the simulations where the protein was constrained to a fix position, the number of ions crossing the membrane is not enough as to compute statistics in the way used for the flexible protein. In order to compute the current, a different approach was used.

A simple model to describe the current evolution from the initial condition to the stationary state is

$$I(t) = I_\infty + (I_0 - I_\infty)\, e^{-t/\tau} \tag{S1}$$

So that the total cumulative current is

$$Q(t) = I_\infty t - \tau\,(I_0 - I_\infty)\left(e^{-t/\tau} - 1\right) \tag{S2}$$

We have fitted the parameters of eq.[S2] with the computed values of $Q(t)$ for times each $\Delta t = 15$ ns. While I_0 and τ depend on the initial condition, it can be expected, close enough to the steady state, that I_∞ is the asymptotic value of the current. To test the model, I_∞ has been computed for the flexible protein and compared to the averaged current between 190 and 230 crossings. As shown in figure S4 the results are quite similar between these two methods. It can be observed that the current saturates very quickly going to a value close to zero. We believe that since the flexible-non-fixed protein vibrates and travels along the membrane, it transmits energy to the particles inside the pore and prevents clogging. This is not the case for a rigid fixed protein. The dynamics of the protein seems then essential to simulate the translocation process.

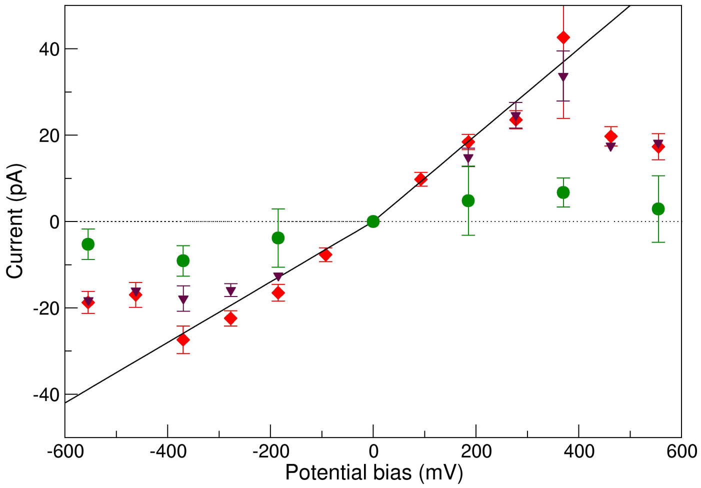

Figure S4. IV curve for flexible protein and rigid fixed protein. Position restrained protein and time statistics (circles), flexible protein and time statistics (diamonds) and flexible protein with crossing statistics (triangles). The black solid line is the reference experimental current, as defined in the main text, divided by a factor of 10.
